# Malignant mesothelioma: Canadian perspective and research directions

**Published:** 2008-04

**Authors:** C.W. Lee, J. Martin, R. MacRae, M.S. Tsao, E. Nguyen, M. Johnston, P. Baas, S. Laurie, R. Feld, N. Murray, F.A. Shepherd

**Affiliations:** * BC Cancer Agency – Fraser Valley Centre, Surrey, BC; † entre Hospitalier de l’Université de Montréal, Montreal, QC; ‡ The Ottawa Hospital Regional Cancer Centre, Ottawa, ON; § University Health Network–Princess Margaret Hospital, Toronto, ON; || University of Toronto, Toronto, ON; # QE II Health Sciences Centre, Halifax, NS; ** The Netherlands Cancer Institute, Amsterdam, Netherlands; †† BC Cancer Agency–Vancouver Centre, Vancouver, BC

**Keywords:** Asbestos, chemotherapy, mesothelioma, pathology, radiology, radiotherapy, surgery

## Abstract

Since the 1960s, the incidence of malignant mesothelioma in Canada has increased dramatically because of work-related asbestos exposures. Treatment options are limited. Although chemotherapy is now an accepted standard in the management of advanced disease, uncertainty surrounds the roles of radical surgery and radiation. In March 2007, a symposium was held in Vancouver, B.C., to review the current approach to malignant mesothelioma in Canada and to discuss development of a national clinical research strategy.

## 1. INTRODUCTION

Since the 1960s, industrial exposure to asbestos has led to a dramatic rise in the number of cases of malignant mesothelioma 1,2. In developed countries, the incidence of mesothelioma is expected to plateau as a result of the introduction of safety guidelines in the workplace since the 1970s 3,4. Bans on asbestos are also anticipated to have a positive effect in countries that have implemented such restrictions 5. However, global production of asbestos has not changed since the late 1990s because of exports to emerging markets. Inadequate management practices and unregulated handling of asbestos in developing countries are expected to sustain the increase in worldwide incidence indefinitely 1.

In Canada, a nationwide audit of all “fatal malignant mesothelial tumors” from 1959 to mid-1968 identified a total of 165 cases 6. The most recent data available from the National Cancer Institute of Canada (ncic) indicate that 391 new cases of mesothelioma were diagnosed in 2003, with 343 deaths from mesothelioma occurring that year 7. Mesothelioma is likely a significant contributor to asbestos-related deaths in Canada, in 2005 accounting for 61% of deaths from occupational diseases and for 31% of all workplace fatalities 8.

The most notable advance in the management of mesothelioma in recent years has been the establishment of platinum-based combination chemotherapy as a standard of care in unresectable disease 9. However, the survival benefit is modest, and uncertainty remains about the roles of radical surgery and radiotherapy 10,11. Further progress in the treatment of mesothelioma is needed. To encourage a national dialogue, a symposium was held in Vancouver, B.C., on March 24 and 25, 2007, sponsored by the ncic Clinical Trials Group (ctg) and the Canadian Institutes of Health Research– Institute of Cancer Research. Current standards in management of mesothelioma were reviewed, and directions for clinical research in Canada were discussed.

## 2. SUMMARY OF PRESENTATIONS

### 2.1 Histology and Molecular Markers

The *Classification for Tumours of the Lung, Pleura, Thymus and Heart* from the World Health Organization categorizes tumours of the pleura into mesothelial tumours and lymphoproliferative disorders 12. Diffuse mesotheliomas comprise the main subdivision of malignant mesothelial tumours, and these are further subcategorized according to histology as epithelial, spindled (sarcomatoid), or biphasic. The *International Classification of Diseases for Oncology* includes corresponding codes for those histologies, together with a code for malignant mesothelioma not otherwise specified 13.

The histologic characteristics of most mesothelioma cells include uniform, bland-appearing, round nuclei with finely dispersed chromatin and a single nucleolus. Occasional cases show greater pleomorphism. A key feature of malignant mesothelial cell proliferation is invasion into adjacent tissue, particularly adipose tissue. Because of an inability to provide this detail, cytology is often inadequate, and biopsy specimens are preferred 14. Still, several histologic features of mesothelioma are shared by other malignancies, which means that making the diagnosis can be challenging.

Routine pathology examination of suspected cases includes immunohistochemistry, although no single tumour marker is considered pathognomonic 15. Table I lists typical marker patterns for epithelial mesothelioma. In practice, a diagnosis of mesothelioma requires two positive markers and two negative markers. In general, immunostains are less helpful in distinguishing sarcomatoid mesothelioma from other sarcomatous neoplasms, although most sarcomatoid mesotheliomas stain positive for broad-spectrum cytokeratin cocktail 16.

Ultrastructural findings can be diagnostic 17. Long microvilli on the cell surface are typical of mesothelioma, and electron microscopy is also of value if it can rule out the diagnosis by revealing features of other malignancies.

Molecular characterization of mesothelioma is at a relatively early stage of development. A number of chromosomal abnormalities have been associated with the disease and its prognosis 12. Inactivation of the *CDKN2A*/*ARF* locus on the short arm of chromosome 9 commonly occurs 18,19. Microarray profiling has also suggested gene patterns associated with prognosis, but validation of results to date are required, and the utility of the observed patterns in a diagnostic algorithm remains uncertain 20.

Perhaps of more immediate clinical interest are studies of serum markers such as mesothelin and osteopontin 21,22. Although not specific to mesothelioma, these molecules appear to be sensitive markers of the disease, and ongoing studies will better define their role in screening for and diagnosing mesothelioma.

### 2.2 Diagnostic and Functional Imaging

The classic anatomic appearance of malignant pleural mesothelioma (mpm) is a tumour rind that follows the contour of the inner chest wall to encircle the lung, in association with a pleural effusion that can be large or small. The pleural thickening is often nodular and involves the mediastinal pleura and fissures. The thickening can be diffuse or focal and associated with ipsilateral volume contraction or expansion. Rarely, mpm will present as a spontaneous pneumothorax without appreciable pleural thickening on computed tomography (ct), or as bilateral disease.

Chest radiography has major limitations when assessing pleural-based disease. Its limited contrast resolution as compared with ct makes areas of solid tumour difficult to distinguish from loculated pleural effusions. The primary imaging modality used to assess the pleura is ct, and criteria have been established to aid in the distinction between benign and malignant pleural processes in ct imaging 23. Although those criteria are not specific to mpm, ct is readily relied on to evaluate and document the extent of disease 24,25.

In assessing response to treatment, the standard Response Evaluation Criteria in Solid Tumors (recist) are difficult to apply, because interpreting the maximum diameter of pleural-based tumour is highly observerdependent and may not accurately reflect the status of the disease 26. Modified response criteria (modified recist) focusing on measuring tumour thickness have been developed that appear better suited to dealing with the measurement problems created by a tumour rind 27,28. More refined assessments of tumour bulk can be obtained using ct imaging and a computer interface to map areas of tumour thickness either by hand or by computer 29. However, the various methods employed produced significant variability, and the technique requires further development. The appropriateness of using, in routine practice, any tumour response criteria developed for clinical trials has not been evaluated.

Magnetic resonance (mr) imaging may not provide a significant advantage over ct imaging in routine staging 30, but it may be of value in individuals who are being considered for radical surgery 31. Of particular benefit is the ability of mr imaging to assess the extent of involvement of mediastinal structures such as the heart (using cardiac-gated sequences), the chest wall, and the diaphragm. Involvement of those structures has implications with respect to surgical approaches and resectability.

Studies involving positron-emission tomography (pet) have demonstrated that mesothelioma is fluorodeoxyglucose (fdg)–avid 32. However, a role for pet imaging in routine assessment has not been clearly defined. Its limited spatial resolution requires integration with ct imaging to accurately localize areas of fdg-uptake 33. In addition, pet imaging is not reliable in identifying the local extent of tumour and mediastinal lymph node involvement, and the latter can be confused with nodular pleural thickening adjacent to the mediastinum 34. Currently, the main use for pet imaging would seem to be to exclude radical surgery for patients with more extensive disease than is apparent with conventional diagnostic imaging 35,36. Other potential uses are in directing biopsy of fdg-avid tumour sites and in re-staging after treatment.

### 2.3 Radical Surgery: Extrapleural Pneumonectomy and Pleurectomy

The role of radical surgery in the management of mpm is a topic of longstanding debate 37. The risks of perioperative morbidity and mortality are significant, and they depend greatly on the experience of the surgical team.

Extrapleural pneumonectomy (epp) entails extrapleural dissection and *en bloc* resection of the pleura and lung, and resection and reconstruction of the pericardium and diaphragm. Reported mortality rates have been as high as 31% 38, although contemporary figures for high-volume centres are 3%–5% 39,40. Postoperative morbidity is more than 50%. Atrial fibrillation is most common, but other serious complications include thromboembolism and acute respiratory distress syndrome.

Pleurectomy involves debulking of the tumour and preservation of the lung. The diaphragm and pericardium are resected as needed. The risk of complications is not as significant as with epp, mortality rates being 1%–2% 39,41.

The survival benefits with radical surgery rely on several factors. The extent to which tumour is debulked is a strong predictor of survival 42,43, as is mediastinal lymph node involvement 44,45.

An assessment of the effect of patient selection on long-term outcomes is not possible 46. Typical candidates for radical surgery have a good performance status, adequate cardiac and pulmonary reserves, and normal hepatic and renal function. Preoperative staging is rigorous, and aside from ct, mr, and pet imaging, mediastinoscopy and laparoscopy are often performed to exclude mediastinal lymph node involvement and extension of disease through the diaphragm 47.

Gauging the effects of induction or adjuvant therapy on survival from published reports is also difficult. There is little expectation that surgery will result in clear resection margins, and locoregional recurrence is a problem with pleurectomy in particular 48. Even with what might be viewed as optimal local disease management, epp followed by hemithorax radiation, the risk of distant relapse is high 49. There is consensus that, if radical surgery is performed, chemotherapy and radical radiation are necessary to deal with macroscopic and microscopic residual disease.

### 2.4 Radiotherapy

There is a limited role for radiotherapy in management of mpm. Palliation of symptomatic chest wall disease is probably the most common indication for radiotherapy; a variety of doses and dose schedules have been reported to achieve pain relief 50.

Prophylactic radiotherapy to sites of chest-wall instrumentation is of less clear benefit. Although mesothelioma has a tendency to track along biopsy and chest-tube tracts, the frequency with which such spread occurs is highly variable; it may depend on the extent of the intervention 51. Reports conflict as to the effectiveness of prophylactic radiotherapy 52–54, which is reflected in guidelines both for 55,56 and against 11 its routine prescription.

With a high expectation of residual disease following radical surgery, many practitioners feel that, to improve local disease control rates, hemithorax radiation is a necessary component of any combinedmodality therapy program. Although the merits of radical surgery are under debate, optimization of radiation doses and application of newer techniques such as intensity-modulated radiotherapy to reduce the risk of local recurrence are attracting interest 57,58. Radiation treatment fields following radical surgery are reasonably well defined by the limits of the resection, but close collaboration with thoracic surgery is necessary to ensure appropriate marking of visible residual disease, the reconstructed diaphragm, and anteromedial pleural reflection. Rigorous definition of the clinical target volume and avoidance of the remaining lung is crucial in minimizing the complications seen with postoperative radiation 59.

### 2.5 Systemic Therapy

Most chemotherapy drugs have limited activity against mpm when tested as single agents 60. However, a landmark study comparing cisplatin plus pemetrexed with cisplatin alone demonstrated a survival advantage for the two-drug regimen 61. Comparable results were seen in a trial of cisplatin plus raltitrexed versus cisplatin 62. As a result, platinum-based chemotherapy is now considered a standard of care in the management of individuals with advanced mpm 9,63. However, the survival benefit with combination chemotherapy is modest, and interest in evaluating new drugs in mpm remains.

Ranpirnase is a ribonuclease with some activity in mpm 64, but the results of a randomized trial combining ranpirnase with chemotherapy are pending. Oral tyrosine kinase inhibitors of epidermal growth factor receptor 65,66 and platelet-derived growth factor receptor 67 have not demonstrated significant activity in mpm, but a monoclonal antibody against the platelet-derived growth factor receptor may be promising 68. Studies of drugs targeting vascular endothelial growth factor and its receptor have so far proven disappointing 69,70, but trials that focus on that pathway are still ongoing, including a phase ii trial of the oral tyrosine kinase inhibitor sunitinib, sponsored by the ncic ctg 71. Two other agents of interest currently undergoing evaluation in large clinical trials are inhibitors of proteasomes 72 and histone deacetylase 73.

### 2.6 Prognostic Factors

Stage and prognostic index scores are both of interest in clinical trials, but they have somewhat variable roles in routine clinical practice.

#### 2.6.1 Staging

A variety of staging systems have been developed based primarily on surgical series; these address the resectability of mpm 38,74. Factors such as the extent of pleural involvement and regional lymph node metastasis are common features associated with survival. However, as a practical matter, complete staging depends on findings at surgery, which many patients do not undergo.

The staging system proposed by the International Mesothelioma Interest Group is generally employed at the present time 75, and ct or mr imaging findings (or both) are used as reasonable approximations for absent surgical data.

#### 2.6.2 Prognostic Indices

Perhaps of equal value are the prognostic indices that have been derived using data from trials performed by the European Organisation for Research and Treatment of Cancer (eortc) and Cancer and Leukemia Group B 76,77. Both indices have been independently validated in other series 78,79, and stratification is based on combinations of biometric data. The eortc index places patients only into good-prognosis and poor-prognosis categories, but that index is easier to calculate than is the Cancer and Leukemia Group B index, which identifies six prognostic groups. Interestingly, neither index depends on stage, despite Butchart staging information having been included in the initial data analyses.

### 2.7 Outcome Measures

#### 2.7.1 Quality of Life

Overall survival is the primary outcome of interest for clinicians treating mpm, but relieving disease-related symptoms and improving quality of life are also viewed as important endpoints in the evaluation of new therapies and interventions 80.

The randomized trials of cisplatin combined with pemetrexed and raltitrexed demonstrated improvements in quality of life, which was assessed using standardized instruments 81,82. The pemetrexed trial used a modified version of the Lung Cancer Symptom Scale (lcss), dubbed the lcss-Meso, which underwent formal validation in patients with mpm 83,84. The raltitrexed trial used the eortc core quality of life questionnaire, the eortc QLQ-C30 85, and the lung cancer module, the eortc QLQ–LC13 86, based in part on the general applicability of the instruments to individuals with advanced malignancies and the commonality of symptoms affecting those with mpm and lung cancer. Another study had demonstrated the validity of the eortc instruments in a small cohort of patients with mpm 87.

#### 2.7.2 Pulmonary Function

Malignant pleural mesothelioma causes a restrictive ventilatory defect 88, classically demonstrated by a markedly diminished total lung capacity with a normal ratio of forced expiratory volume to vital capacity on pulmonary function testing 89.

Response of mpm to treatment is associated with significant improvements in forced expiratory volume and vital capacity, and pulmonary function correlates with the bulk of disease 27,90,91.

#### 2.7.3 Circulating Tumour Markers

Soluble mesothelin-related proteins (smrps) include mesothelin, megakaryocyte potentiating factor, and any other related soluble molecules that are bound by the monoclonal antibody OV569 92. Levels of smrps are notably elevated in serum samples from individuals with mpm, appear to be correlated with the bulk of disease, and may be a useful prognostic marker 21,93,94. However, these relationships may hold true only in individuals with epithelioid, and not sarcomatoid, mesothelioma.

Serum osteopontin levels are also high in individuals with mpm and may be useful in diagnosing the disease in at-risk populations 22. Osteopontin levels are prognostic, but as compared with levels of smrps, their specificity is limited 93,95.

Levels of smrps fall after surgery for mpm 21, but the data are currently insufficient to recommend routine use of either smrps or osteopontin in monitoring response of mpm to treatment.

### 2.8 Clinical Research Directions: National Strategy

The Symposium on Malignant Mesothelioma: Canadian Perspective and Research Directions was held in March 2007 and was attended by 52 physicians and researchers from across the country. The specialties represented included thoracic surgery, pathology, radiology, radiation oncology, and medical oncology. After presentations on the diagnosis and management of mpm, the attendees took part in an open discussion on issues related to the development of a national research strategy.

The frequency of mpm and the availability of specialist resources across the country place some limitations on the scope of clinical research. A large randomized controlled trial to evaluate the role of a novel therapy or intervention cannot reasonably be performed within Canada alone; international collaboration is required. In particular, the surgical expertise capable of carrying out a trial of combined-modality therapy is restricted to a handful of centres across the country. With the uncertainties and biases related to radical surgery among thoracic surgeons, performance of a trial with randomization to a particular surgical procedure is not considered practicable.

Similarly, questions related to hemithorax radiation are relevant only to the small number of centres with experience in delivering combined-modality therapy. Although the value of prophylactic radiotherapy to sites of chest wall instrumentation is somewhat controversial, and few, if any, restrictions would affect the ability of centres to participate, performing another trial to try to resolve this issue is considered lowpriority. Other concepts for trials of radiotherapy were not put forward.

Continued work in the area of new drug development received strong support. The recent opening of the phase ii trial of sunitinib has demonstrated the feasibility of, and interest in, performing such studies. The impetus to develop and evaluate novel targeted anticancer agents is as relevant to mpm as it is to other malignancies, and mechanisms such as the ncic ctg exist for carrying out such work in Canada.

To facilitate clinical trials, standardization of diagnostic and functional imaging procedures for staging is recommended (see Table ii). Routine clinical data collection to include information to calculate prognostic index scores was also advised.

In evaluating the effects of new treatments and interventions, overall survival and quality of life were felt to be the most important outcome measures. Assessment of tumour response with serial ct imaging using the modified response criteria developed for mpm was advocated. Because of a strong correlation with tumour response, incorporation of pulmonary function testing as a trial endpoint was not considered necessary unless a specific question regarding lung function arises.

With the typical requirement for tissue biopsy specimens to diagnose mpm, there was considerable support for correlative tissue studies to advance understanding of the biology of mesothelioma. Development of a national mesothelioma tissue registry was recommended, with links to Canadian expertise in genomics and proteomics.

Areas of research that were not discussed were ideas for studies of screening at-risk populations and early detection, development of novel imaging modalities, and general issues of palliative and supportive care. A second symposium in 2–3 years is warranted to tackle those issues and to review the progress that will have been made in the intervening period.

## 3. CONCLUSIONS

With the expectation that the incidence of mpm will remain unchanged—if not increase—over the next decade, a national research strategy is needed. Questions regarding combined-modality therapy can be addressed only at centres with appropriate surgical and radiation oncology expertise, and those questions will tend to focus on institutional experiences. Aside from continuing to engage in new drug development, efforts will be made to establish a national mesothelioma tissue registry to support basic and clinical research. Study of other issues related to screening, diagnosis, and overall patient care requires further discussion involving researchers and health care professionals, as well as patients with mpm and their families.

## Figures and Tables

**TABLE I:** Immunohistochemical markers of epithelioid mesothelioma

*Positive*	*Negative*
Calretinin	TTF-1
CK5/6	CEA
WT-1	BerEP4
D2-40	CD15 B72.3

**TABLE II:** Staging of mpm with diagnostic and functional imaging

Imaging modality	Recommendation
Computed tomography	Routine for all patients
Magnetic resonance imaging	Not routine; potential value in preoperative assessment of extent of involvement of diaphragm and mediastinum
Positron-emission tomography	Not routine; potential value in preoperative assessment
